# Gilteritinib in an Isolated CNS Recurrence of FLT3‐ITD Positive AML

**DOI:** 10.1002/ccr3.71541

**Published:** 2026-01-16

**Authors:** Lina Susana Silva‐Bermudez, Alexander Streuer, Foteini Christodoulou, Philipp Eisele, Laurenz Steiner, Georgia Metzgeroth, Michael Platten, Wolf‐Karsten Hofmann, Mohammed L. Abba

**Affiliations:** ^1^ Institute of Transfusion Medicine and Immunology, Medical Faculty Mannheim Heidelberg University Mannheim Germany; ^2^ Department of Hematology and Medical Oncology, Medical Faculty Mannheim Heidelberg University Mannheim Germany; ^3^ Department of Neurology, Medical Faculty Mannheim and Mannheim Center of Translational Neurosciences (MCTN) Heidelberg University Mannheim Germany

**Keywords:** AML, CNS relapse, FLT3 rearrangements, gilteritinib

## Abstract

Central nervous system (CNS) involvement in adult acute myeloid leukemia (AML) is uncommon and more frequently observed at relapse than at initial presentation. Typically, CNS relapse coincides with systemic disease, evidenced by detectable blasts in the bone marrow (BM) and/or peripheral blood. We describe a rare case of an 82‐year‐old patient with a history of AML, diagnosed three and a half years earlier, who developed an isolated CNS relapse while receiving palliative chemotherapy—without concurrent BM involvement or molecular evidence of systemic progression. Cerebrospinal fluid (CSF) analysis revealed blast cells harboring an FLT3 internal tandem duplication (FLT3‐ITD). Targeted therapy with an FLT3 tyrosine kinase inhibitor (TKI) achieved complete remission, with clearance of FLT3‐ITD from the CSF.

## Summary

This case demonstrates that an isolated CNS relapse can occur in acute myeloid leukemia patients who are otherwise in complete hematological and molecular remission, and that targeted therapy, where applicable, as in our case against FLT3, can result in renewed remission.

## Graphical abstract

Time course showing changes in cell count and FLT3‐ITD ratio in cerebrospinal fluid (CSF) from diagnosis through treatment.

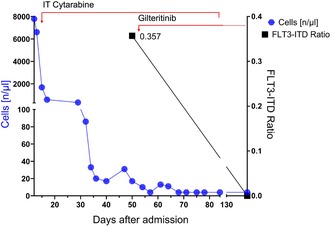

## 1 Introduction

Acute myeloid leukemia (AML) with central nervous system (CNS) dissemination is rare in adult patients at diagnosis. Several studies report an estimated frequency of 0.6%–1% for meningeal involvement as an initial manifestation of AML [1, 2]. Involvement of the parenchyma is even less common [1]. A large retrospective study of more than 3000 AML patients who underwent lumbar puncture (LP) at the time of diagnosis, regardless of neurological symptoms, also showed a low frequency of 0.86% [2].

Meningeal relapse may occur in up to 3% of patients [3]. Less frequently, is an isolated CNS manifestation in the absence of active hematologic disease in the bone marrow (BM) or in peripheral blood, with an incidence of approximately 1% [4]. This scenario has been associated with younger age and high white blood cell (WBC) count [4]. There has been a positive association between FLT3 internal tandem duplication (ITD) and CNS involvement [5, 6]. In addition, monoblastic/monocytic (FAB M5) morphology and complex karyotypes, such as trisomy of chromosomes 8 and 22, have also been associated with CNS compromise [3, 4].

Indeed, CNS involvement should be suspected whenever AML patients develop new neurologic symptoms. However, CNS spread may also be asymptomatic. If symptomatic, it can manifest as headaches, indicating increased intracranial pressure, altered mental status, or radicular pain in the case of spinal cord compression. When suspected, diagnostic strategies include flow cytometry, immunophenotyping, and microscopic analysis of cerebrospinal fluid (CSF). The severity of symptoms may not always be related to the number of blasts in the CSF. Magnetic resonance imaging (MRI) and computed tomography (CT) scans are additional diagnostic tools that could be used to assess CNS infiltration [7].

The incidence of CNS relapse in patients with AML and CNS infiltration at initial diagnosis is reported to be 12% [8]. There is conflicting evidence regarding the overall survival in these patients. However, most studies report a decreased overall survival compared to patients without CNS compromise. After treatment, the overall survival is approximately 25% at 3 years [8].

Standard treatment for meningeal infiltration includes the intrathecal administration of methotrexate, cytarabine, and glucocorticoids (NCCN Guidelines 2024). In most cases, intrathecal chemotherapy (IT) is effective in clearing blasts and improving neurologic symptoms [9].

Tyrosine kinase inhibitors (TKI) are usually given as add‐on therapy when targetable mutations exist. Gilteritinib is a second‐generation selective FLT3 TKI targeting FLT3‐ITD and FLT3‐TKD mutations [10]. This drug is now the gold standard for refractory FLT3‐mutated AML due to its efficacy in improving overall survival compared to chemotherapy (9.3 vs. 5.6 months) [11]. There is no explicit evidence regarding the use of gilteritinib in cases of CNS involvement, other than a few case reports [12, 13]. In addition, the pharmacokinetics of this drug in the CNS requires further investigation [14]. Still, the fact that it is a small, lipophilic, highly tissue‐distributed molecule with a long half‐life, and is not completely excluded by efflux transporters at the blood–brain barrier may partly explain its CNS activity [15].

Here, we present a case of the successful treatment of an FLT3‐mutated AML with isolated CNS relapse, while in hematological and molecular remission in BM, using gilteritinib. The patient provided written informed consent for publication of the medical case details.

## 2 Case History

An 82‐year‐old male patient was diagnosed with AML with a NPM1 mutation three and a half years ago. The karyotype was normal, and additional mutations were detected in DNMT3A (VAF 48%), FLT3‐ITD (VAF 3%), NPM1 (VAF 23%), and SH2B3 (VAF 47%). The initial BM blast count was 73%. Intensive chemotherapy with curative intent was not feasible due to the patient's age, reduced performance status, and comorbidities. The patient was started on a combination of the demethylating agent decitabine (DEC, 20 mg/m^2^, d1–5) and the Bcl‐2 inhibitor venetoclax (VEN, 100 mg/day, d1–27), according to standard protocols. The reduced dose of 100 mg was due to the concomitant use of posaconazole.

A biopsy performed 3 weeks after the initiation of treatment showed less than 5% blasts in the BM with a completely negative leukemia‐associated immunophenotype (LAIP). The treatment was continued, and another BM biopsy was performed after cycle 6. A complete remission with incomplete count recovery (CRi) was confirmed with a BM blast count of < 5%, no circulating blasts, platelet count ≥ 100 × 10^9^/L (150 × 10^9^/L), and a residual neutropenia < 1.0 × 10^9^/L (0.04 × 10^9^/L), which recovered over time reaching ≥ 0.5 × 10^9^/L 5 days later (0.59 × 10^9^/L, 5 days later 3.07 × 10^9^/L). All previously described mutations were no longer detected.

In order to maintain remission, the patient was treated further with a total of 22 cycles of combined therapy with decitabine and venetoclax. However, treatment courses were at times delayed depending on the patient's prevailing clinical condition and the peripheral blood results. The patient was in documented complete morphological and molecular BM remission for 33 months. A BM biopsy performed 1 year after the initial diagnosis continued to show remission. Under this regimen, the blood count showed occasional thrombocytopenia but rarely < 100 × 10^9^/L. Only two red blood cell units were required during the early phases of treatment after the initial AML diagnosis. The dose of venetoclax could eventually be increased to 400 mg/day in subsequent cycles. However, due to long periods of therapy‐associated pancytopenia, the total dose of venetoclax was reduced to 200 mg/day. The patient did well on this regimen and continued his treatment further, with regular check‐ups between treatment cycles.

**FIGURE 1:**
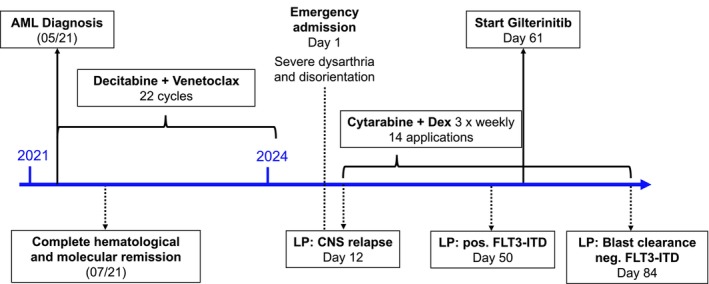
Timeline of the reported case.

Three years into his treatment, the patient was rushed to the emergency department with new‐onset severe dysarthria and disorientation see (Figure 1).

## 3 Investigations and Treatment

The patient was promptly admitted and treated as a suspected case of acute left hemispheric ischemic stroke. The likelihood of this diagnosis was further strengthened by the patient's history of intermittent atrial fibrillation. The initial brain CT scan showed no evidence of ischemia or thrombosis. The patient was treated with i.v. thrombolytics based on the prevailing clinical signs and symptoms. Notably, this led to a transient improvement in his symptoms. Subsequently, repeated focal seizures without impairment of consciousness were observed. The postinterventional CT and MRI scans were normal.

**FIGURE 2:**
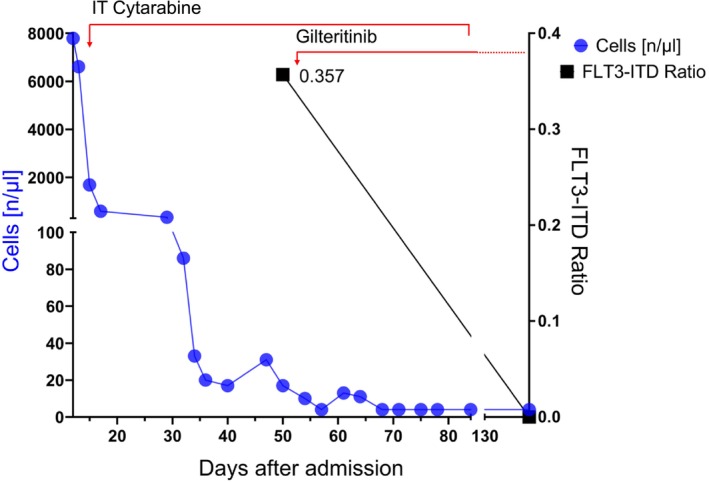
Cerebrospinal fluid (CSF) cell counts in the days following admission. The initial treatment was intrathecal therapy (IT) with cytarabine 40 mg and dexamethasone 4 mg three times a week. A decrease in cell count was observed after IT. The FTL3‐ITD ratio was assessed and decreased significantly under gilteritinib therapy.

**FIGURE 3:**
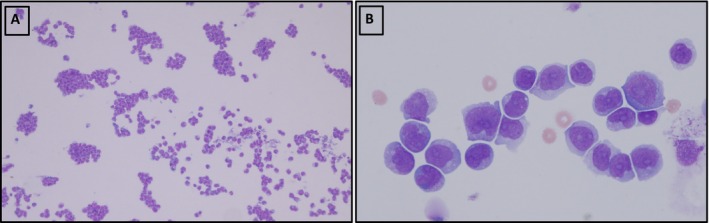
Cytospinslides of cerebrospinal fluid (CSF) stained with May‐Grünwald‐Giemsa (Zeiss Plan‐Apochromat, x10, x63). (A) An overview showing a significant increase in atypical cells, (B) the same sample at a higher magnification, revealing the morphological details of the blast cells, revealing monocytic differentiation, with prominent nucleoli and occasional fine cytoplasmic granulation.

An LP was subsequently carried out, which revealed a cloudy CSF with a cell count of 7790/μL (see Figure 2). A cytological examination of this fluid revealed a cell‐rich specimen with large blast‐like cells (Figure 3), with subsequent immunophenotyping by FACS showing 98% myeloid blasts, confirming a CNS relapse of his AML. To rule out a systemic relapse of the AML, a BM biopsy was performed. This showed a completely blast‐free marrow with no mutations and/or cytogenetic aberrations. Physical examination and CT scan did not reveal any other extramedullary manifestations. Consequently, intrathecal therapy (IT) with cytarabine 40 mg and dexamethasone 4 mg three times weekly was initiated. A remarkable improvement in the patient's symptoms was observed, and the cell count reduced to 13/μL, with a significant reduction of blasts. Since the patient's initial AML phenotype at diagnosis was FLT3‐ITD positive, the CSF was also tested for this rearrangement. This was confirmed to be the case.

## 4 Outcome and Follow‐Up

The patient was discharged home, and the IT application of cytarabine and dexamethasone was continued twice weekly in our outpatient hematology clinic until blast clearance was achieved. Following the confirmation of FLT3‐ITD (Ratio 0.357) in the CSF, the patient was started on oral gilteritinib. The treatment was given at the recommended dose of 120 mg/day. Unfortunately, this resulted in an increase in liver enzymes, and the dose was reduced to 80 mg/day due to suspected hepatotoxicity. ALT/AST levels rose to approximately 20 times the upper limit of normal (ULN) with full‐dose therapy. These values reduced to about 10 times the ULN within 3 weeks of dose reduction and were within normal limits at 12 weeks postdose reduction. ALT values were more elevated than AST.

Ten weeks after the start of this TKI treatment, a repeat LP was performed, which confirmed the absence of cells and no measurable FLT3‐ITD by PCR. The patient remains in remission as of his last visit, 15 months after his CNS relapse and 14 months after starting gilteritinib. The patient is still being followed up. Certainly, long‐term follow‐up is needed to determine the durability of the response. The patient's quality of life also improved substantially, especially considering that the patient had no residual neurological deficits. Moreover, the frequency of hospital visits decreased compared to the time he was on the combination of decitabine and venetoclax, resulting in more quality home time with family.

## 5 Discussion

The evidence for the use of gilteritinib in FLT3‐mutated AML patients with CNS relapse is scarce but promising. There is ample evidence for the use of gilteritinib in the treatment of AML with FLT3 mutations. However, clinical trials have not included cases of CNS involvement, leaving uncertainty about its effect [16]. Few case reports have shown the use of gilteritinib in AML for CNS relapse. Ji et al. reported a case of a woman with FLT3‐mutated AML who was initially treated with gilteritinib and who, after allogeneic stem cell transplantation, developed a CNS relapse without a BM relapse, similar to our case. She was then re‐treated with gilteritinib along with IT in combination with venetoclax, which was stopped after 3 weeks due to suspected toxicity. She continued on gilteritinib and achieved remission [12]. In contrast, in our case, the patient was not transplanted and did not receive venetoclax following the relapse.

Another case was reported by Perrone et al. [13] who treated a patient with AML with hematological and CNS relapse. In this patient, IT was contraindicated due to the presence of a bilateral subdural hematoma together with meningeal sarcoma on MRI. In this constellation, gilteritinib monotherapy was continued, resulting in a reduction in the size of the meningeal lesions and resolution of the myeloid sarcoma [13]. This case highlights the potential of gilteritinib in the treatment of extramedullary relapse in the CNS. However, the control of treatment success in the CSF was not performed.

Vignal et al. [14] demonstrated the presence of active gilteritinib in the CSF and also showed that CSF from patients on gilteritinib therapy cultured with an AML cell line harboring a FLT3‐ITD had an anti‐leukemic effect and reduced proliferation in vitro [14]. This is the first report on the pharmacokinetic effects of this drug in the CNS. This group also presented four cases in which gilteritinib was used in different clinical scenarios, including CNS involvement at AML diagnosis and both hematological and CNS relapse, along with different chemotherapy regimen strategies.

Our report illustrates a successful case of the use of gilteritinib in combination with IT in the treatment of FLT3‐mutated AML CNS relapse without BM involvement. Importantly, CNS blast clearance was achieved with the combination of both intrathecal chemotherapy and gilteritinib.

Based on our and other successful cases, it is reasonable to expand the current clinical evidence regarding the use of gilteritinib in CNS relapse. Four of six cases in the literature show a benefit of gilteritinib in meningeal relapsed AML (see Table 1). It will also be interesting to investigate whether the efficacy of this drug changes with the type of CNS relapse, such as blasts in the CSF or meningeal sarcoma or other more localized extramedullary lesions. Further, it will be necessary to investigate how the penetrance or distribution of gilteritinib changes with the use of other drugs. This could potentially expand the therapeutic options available to patients with FLT3 mutations who are not ideal candidates for conventional chemotherapeutic regimens. Notably, our patient was heavily treated before his relapse, and this, with his advanced age, makes a generalization of his outcome to younger, more therapy naïve patients presumptuous.

**TABLE 1:** Overview of the reported AML cases with CNS relapse treated with gilteritinib.

Author	Diagnosis	CNS relapse manifestation	Symptoms	Treatment	Gilteritinib duration (months)	Remission duration (months)	Complications
Ji 2024	High‐risk, FLT3‐mutated relapsed/refractory	Blasts in CSF, after allogenic stem cell transplantation, no BM involvement	Unilateral tinnitus with decreased vision	5 × 2‐w IT (cytarabine 50 mg + methotrexate 15 mg + dexamethasone 5 mg). VEN (200 mg/day) and gilteritinib (120 mg/day). VEN was paused after 3 w due to pancytopenia and gilteritinib was reduced to 80 mg/day	28, until follow‐up	Until follow‐up	Immune hepatic injury, pancytopenia
Vignal 2023	M1‐AML with NPM1 and FLT3‐ITD mutation	Blasts in CSF, BM involvement	Headache and radicular pain	6 IT (corticosteroid, methotrexate, and cytarabine) until CSF blast cell clearance, followed by cytarabine + gilteritinib (120 mg/day). Long‐term maintenance with gilteritinib and in toto encephalic irradiation	12	12	—
	Hyperleukocytic M5‐AML, NPM1 and FLT3‐ITD mutation	Blasts in CSF, with dermal relapse	Delirium, psychomotor retardation, and an erythematous maculopapular rash	3 IT (corticosteroid and methotrexate) injections + gilteritinib (120 mg/day). Decreased to 80 mg/day due to hepatic toxicity	12, until follow‐up	Until follow‐up	Hepatic toxicity
	M5‐AML with NPM1 and FLT3‐ITD mutation	Blasts in CSF	—	4 IT, followed by gilteritinib (120 mg/day), 1 m after VEN (400 mg/day) was added	11	9	—
	M5‐AML with NPM1 mutation	Blasts in CSF	Cauda equina syndrome	6 IT + gilteritinib (120 mg/day)	13	12	—
Perrone 2021	Therapy‐related‐AML with NPM1 and FLT3‐ITD mutation	Meningeal manifestation with bilateral subdural hematoma, BM involvement	Burning leg pain, weakening of the unilateral hand and leg muscles	Gilteritinib (120 mg/day)	3	Disappearance of myeloid sarcoma but no BM remission	—

For patients not receiving intensive chemotherapy, this is likely to be an important addition to the upfront treatment of FLT3‐mutated AML. Gilteritinib is approved as monotherapy for the treatment of adult patients with relapsed or refractory acute myeloid leukemia (AML) with a FLT3 mutation. Gilteritinib was chosen above midostaurin and the other TKIs because the evidence supporting CNS penetration (case reports and preclinical data) indicates that it is the most promising alternative. Midostaurin has poor CNS penetration and limited activity as monotherapy. Quizartinib and crenolanib have no proven CNS penetration [17]. While gilteritinib's hepatotoxicity necessitated a dose reduction in our patient, tyrosine kinase inhibitors (TKIs) are associated with diverse tissue‐specific risks. For instance, imatinib—a BCR‐ABL1 TKI—has been implicated in spontaneous splenic rupture in CML, possibly due to rapid changes in splenic architecture or vascular integrity during treatment response [18]. Although gilteritinib's CNS efficacy was pivotal here, clinicians must remain vigilant for off‐target effects, particularly in organs prone to infiltration (e.g., spleen, liver, or meninges).

## Author Contributions

**Lina Susana Silva‐Bermudez:** conceptualization, data curation, writing – original draft, writing – review and editing. **Alexander Streuer:** methodology, project administration. **Foteini Christodoulou:** methodology, project administration. **Philipp Eisele:** investigation, methodology, project administration, writing – review and editing. **Laurenz Steiner:** data curation, methodology. **Georgia Metzgeroth:** conceptualization, formal analysis, methodology, project administration, writing – review and editing. **Michael Platten:** project administration, writing – review and editing. **Wolf‐Karsten Hofmann:** project administration, resources, writing – review and editing. **Mohammed L. Abba:** conceptualization, data curation, methodology, writing – original draft, writing – review and editing.

## Funding

The authors have nothing to report.

## Ethics Statement

The research was conducted ethically in accordance with the World Medical Association Declaration of Helsinki.

## Consent

The patient provided written informed consent for data collection. Written informed consent was obtained from the patient for publication of the details of the medical case.

## Conflicts of Interest

The authors declare no conflicts of interest.

## Data Availability

The data that support the findings of this study are available on request from the corresponding author. The data are not publicly available due to privacy or ethical restrictions.
